# Frailty Influence on Postoperative Surgical Site Infections After Surgery for Degenerative Spine Disease and Adult Spine Deformity. Can a Frailty Index be a Valuable Summary Risk Indicator? A Systematic Review and Metanalysis of the Current Literature

**DOI:** 10.1177/21925682241235605

**Published:** 2024-02-21

**Authors:** Marco Manzetti, Alberto Ruffilli, Giovanni Viroli, Matteo Traversari, Marco Ialuna, Francesca Salamanna, Simona Neri, Cesare Faldini

**Affiliations:** 1 1st Orthopaedic and Traumatologic Clinic, 18509 IRCCS Istituto Ortopedico Rizzoli, Bologna, Italy; 2 Department of Biomedical and Neuromotor Science - DIBINEM, University of Bologna, Bologna, Italy; 3 Surgical Science and Technology, 18509 IRCCS Istituto Ortopedico Rizzoli, Bologna, Italy; 4 Medicine and Rheumatology Unit, 18509 IRCCS Istituto Ortopedico Rizzoli, Bologna, Italy

**Keywords:** frailty, spine surgery, infection, degenerative spine, deformity, frailty index

## Abstract

**Study Design:**

Metanalysis.

**Objective:**

Surgical site infections (SSI) is one of the commonest postoperative adverse events after spine surgery. Frailty has been described as a valuable summary risk indicator for SSI in spine surgery. The aim of this metanalysis is to evaluate the influence of frailty on postoperative SSI in this cohort and provide hints on which index can predict the risk of SSI.

**Methods:**

Papers describing the postoperative SSI rate in adult degenerative spine disease or adult spine deformity patients with varying degrees of frailty were included in the analysis. The SSI rate in different grades of frailty was considered for outcome measure. Meta-analysis was performed on studies in whom data regarding patients with different levels of frailty and occurrence of postoperative SSI could be pooled. *P* < .05 was considered significant.

**Results:**

16 studies were included. The frailty prevalence measured using mFI-11 ranged from 3% to 17.9%, these values were inferior to those measured with mFI-5. Significant difference was found between frail and non-frail patients in postoperative SSI rate at metanalysis (z = 5.9547, *P* < .0001 for mFI-5 and z = 3.8334, *P* = .0001 for mFI-11).

**Conclusion:**

This is the first meta-analysis to specifically investigate the impact of frailty, on occurrence of SSI. We found a relevant statistical difference between frail and non-frail patients in SSI occurrence rate. This is a relevant finding, as the ageing of population increases alongside with spine surgery procedures, a better understanding of risk factors may advance our ability to treat patients while minimizing the occurrence of SSI.

## Introduction

The continuous increasing in ageing and life expectancy has slowly led to an increased prevalence of a rising number of elderly patients affected by degenerative spine disease (DSD) and deformity.^1^ This influenced inevitably the preservation of independence and prevention of disability of the elderly, making frailty one of the most studied topics in the recent literature.^2-4^

Frailty is a multidimensional syndrome characterized by reduced reserve or function of multiple physiologic systems, increasing vulnerability and inability to recover homeostasis after a stressor occurrence, such as surgery, falls, diseases and trauma. Thus a frail subject is prone to develop increased dependency or death.^5^

Although frailty could be challenging to diagnose and prone to subjective statement, describe different levels of frailty in an objective manner may help determine which patient may be too high risk of undergoing a surgical procedure. Conversely, a surgical procedure for a disease that hinder a patient’s functional capacity, may enhance patient’s quality of life, and reduce its frailty. The frailty index (FI) and its modifications (modified FI), which reflect the accumulation of comorbidities, or the FRAIL scale, which evaluates the age-associated phenotype of frailty, are only two examples of the many tools that have been published to assess objectively frailty with various designs.^6-8^

Flexman et al.^9^ analyzed a large cohort of patients undergoing different spine surgery procedure, and found a prevalence of frailty of 4% in the total population that doubles to 8% after considering patients older than 65 years. These estimates further increase if considering other cohorts, rising to 59% in adult spine deformity patients^10^ and 80% in metastatic spine tumors.^11^

Higher values of frailty are related to worst postoperative adverse events and mortality after spine surgery, above all in adult spine deformity patients where preoperative frailty assessment is mandatory before considering the invasiveness of a surgical procedure.^12^

Surgical site infections (SSI) is one of the commonest postoperative adverse event after spine surgery, leading to frequent readmissions for debridement surgery, increasing morbidity and healthcare costs.^13,14^ Koutsombelis et al.^15^ studying a large cohort of posterior lumbar instrumented patients, identified surgical and patient-related risk factors of postoperative SSI. Among them, frailty have been strongly linked to postoperative complications and mortality.

In the recent years, various frailty indices have been investigated as a valuable summary risk indicator for SSI in spine surgery.^16-19^ However, discordant mixed results have been obtained for various indices, suggesting that additional work is needed to better elucidate the relationship between postoperative complications and baseline frailty in spine surgery.^20-24^

To the best of the Authors’ knowledge, there are currently no metanalysis that analyze the impact of frailty on the rate of SSI after surgery for DSD and ASD. These two pathologies share similar characteristics seen that degenerative process of the spine ultimately can cause sagittal and coronal imbalance, leading to structural deformities and poor health related quality of life,^25,26^ and typically occur in the elderly and frail population. Frailty has been described as an independent risk factor for SSI and other complications, regardless of surgical procedure, operative time, or invasiveness of the operation.

Thus, the aim is to evaluate the influence of frailty on the postoperative infection rate in this cohort and provide hints on which index can predict the risk of SSI.

## Materials and Methods

### Review Design

A systematic review of the literature regarding SSI after spine surgery for DSD and ASD was carried out following the Preferred Reporting Items for Systematic Reviews and Meta-Analyses (PRISMA guidelines).^27^

The Oxford level of evidence scale^28^ was used to assess the level of evidence of the included studies (full version for randomized and non-randomized clinical trials, modified version for all other studies). Inclusion criteria were considered: papers describing the SSI rate of elective thoracolumbar and lumbar spine procedures in DSD or ASD patients with varying degrees of frailty. Frailty was assessed using indices derived from Rockwood’s accumulation of deficit model, including the mFI-11, mFI-5, ASD-FI, and HRFS. These indices characterize different levels of frailty based on the increasing number of variables (such as pathologies or deficits) present in the patient. For instance, a patient scoring 0 on the mFI-5 is considered non-fragile, a score of 1 indicates mild to moderate frailty, and a score of 2 signifies severe frailty.

Exclusion criteria were applied: Isolated case reports/series with less than 5 patients, technical notes, expert opinions, literature reviews, meta-analysis, biomechanical and/or in vitro studies; papers providing incomplete data or not providing data regarding SSI or frailty rate, papers describing outcomes in cervical spine, tumoral or metastatic spine, trauma, infection and revision surgery. Papers reporting results in patients with diagnosis of rheumatologic disease, connective tissue disease and malabsorptive disorder were also excluded, because these conditions can affect the bone and muscles quality confounding the results.

Studies not indicating either the DSD or ASD diagnosis, but excluding patients undergoing cervical spine, tumoral or metastatic spine, trauma, infection and revision surgery were also considered for inclusion.

Articles in English on peer-reviewed journals who met the population, intervention, comparison, and outcomes criteria on systematic reviews were considered for inclusion.

Randomized controlled trials, prospective and retrospective cohort studies, and case series (CS) were considered for inclusion.

### Search Strategy

An electronic systematic search of the available English literature on three large electronic databases (Pubmed-MEDLINE, Scopus and Google Scholar) was performed over the years 2014-2023 to identify eligible studies. The online literature search was conducted in August 2023 by two authors (MM, and MT). The authors stated the following research questions: “*Can the assessment of frailty status predict the risk of surgical site infection after ASD and DSD surgery in adults?*” *Which index predicts the most the risk of surgical site infection? Is frailty a factor we need to consider before spine surgery?*”

The search was conducted using combinations of the following keywords: “*Frailty*”, “*degenerative spine disease*”, “*adult spine deformity*”, “*lumbar*”, *“thoracic”,* “*thoracolumbar*”, “*elective*” “*infection*”, “*surgical site infection*”, “*frailty index*”, “*modified frailty index*”, “*mFI-11*”, “*mFI-5*”, *“ASD-FI”,* “*outcomes*”, “*complications*”, “*elderly*”, “*thoracolumbar fusion*” and “*lumbar fusion*”.

### Study Selection

**Figure 1.**
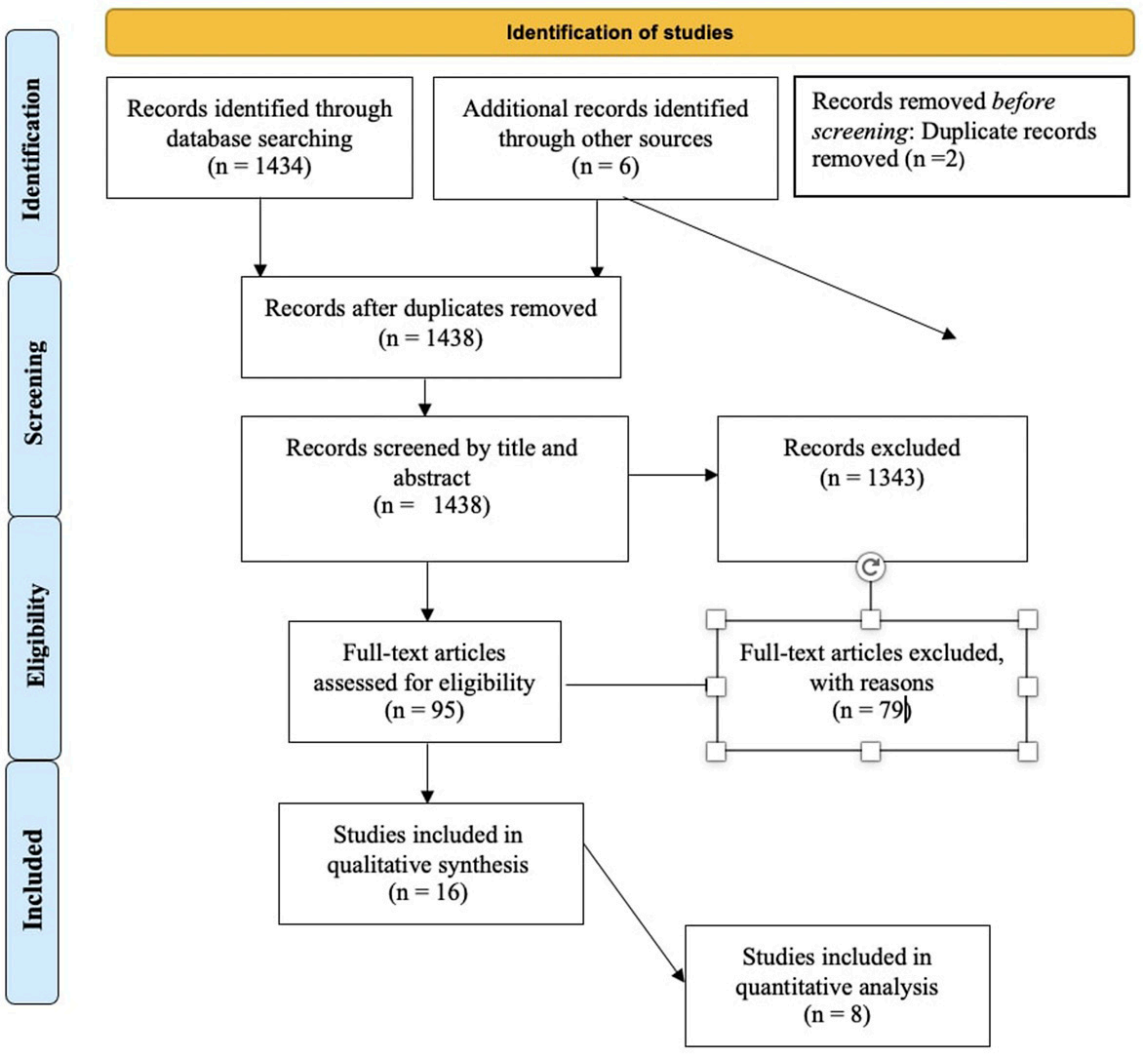
Prisma flow diagram of the included studies.

After screening the titles and abstracts, the full-text articles were obtained and reviewed. A manual search of the bibliography of each of the relevant articles was also performed to identify potentially missed eligible papers. Reviews and meta‐analyses were also analyzed to potentially broaden the search for studies that might have been missed through the electronic search. Duplicates were removed. The study selection process carried out in accordance with the PRISMA flowchart^27^ (Figure 1). The present systematic review was accepted for registration in the PROSPERO database for systematic reviews^29^ (ID: CRD42023465550).

### Data Extraction

Two authors (MM and MT) extracted the data through a standardized data collection form. Three authors (MM, GV and MT) checked the data for accuracy, and inconsistent results were discussed. Data concerning study design, number and demographics of patients, cohort studied, frailty evaluation tool, frailty prevalence, and results were extracted and summarized in Table 1.

**Table 1.** Details of the Included Studies, NS = Not Specified, mFI-11 = Modified Frailty Index 11 Elements, ASD-FI = Adult Spine Deformity Frailty Index, m-ASD-FI = Modified Adult Spine Deformity Frailty Index, mFI-5 = Modified Frailty Index 5 Elements, NSQIP = National Surgical Quality Improvement Project, ISSG = International Spine Study Group, NRD = Nationwide Readmission Database.

Authors (year, Journal)	Study design (level of evidence)	Number of patients (M: F or range)	Mean age (± or range) (years)	Cohort	Frailty evaluation tool	Frailty prevalence	Results
D.M. Leven (2016, Spine)	Retrospective study of prospectively collected data (III)	1001 (461:540)	59.2±14.1	NSQIP database (years 2005-2012)	mFI-11	mFI-11 = 0: 389 (39 %) mFI-11 = .09: 451 (45%) mFI-11 = .18: 131 (13%) mFI-11≥.27: 30 (3%)	As the mFI increased from 0 to >.29 wound complications increased from 3% to 10%. This suggests a large percentage of early 30-day reoperations were caused by wound infections and a clear association with increasing mFI scores.
D. M. Leven (2017, global spine Journal)	Retrospective study of prospectively collected data (III)	6094 (2742:3352)	60±13.9 (18-90)	NSQIP database (2005-2012)	mFI-11	mFI-11 = 0: 2266 (37.2%) mFI-11 = .09: 2309 (37.9%) mFI-11 = .18: 1147 (18.8%) mFI-1 = .27: 295 (4.8%) mFI-11≥.36: 77 (1.3%)	The rate of wound complications all increased in a stepwise fashion with increasing of mFI (*P* < .02)
E.K. Miller (2017, neurosurgial Focus)	Retrospective analysis of prospectively collected database (III)	417 (82:335)	Non-frail: 49±1.3 Frail: 61±1 Severely frail: 63±1.1	ISSG database (2010-2014)	ASD-FI	Non-frail (ASD-FI<.3): 171 (41%) Frail (ASD-FI 0.3-.5): 162 (38.8%) Severely frail (ASD-FI>.5): 84 (20.2%)	On univariate analysis, the severely frail patients had higher odds of wound dehiscence (OR 11.95% CI 1.1-3.7) and deep wound infection (OR 4.3 95% CI 1.0-18). Moreover, on multivariate analysis, the severely frail patients also have higher odds of experiencing wound dehiscence (OR 13.4, 95% CI 1.5-120), and deep wound infection (OR 8.0, 95% CI 1.3-49).
E.K. Miller (2018, European spine Journal)	Retrospective analysis of prospectively collected database (III)	266 (63:203)	Non-frail: 43±1.7 Frail: 57±1.8 Severely frail: 62±2.6	European spine study group database (2012-2013)	ASD-FI	Non-frail (ASD-FI<.3): 135(50.8%) Frail (ASD-FI 0.3-.5): 90 (33.8%) Severely frail (ASD-FI>.5): 41 (15.4%)	On univariate analysis, compared to non-frail patients, severely frail patients had higher odds of developing a wound infection (OR 8.4; 95% CI 1.6-7.6) On multivariate logistic analysis, severely frail patients had higher odds of wound infections (OR 9.7; 95% CI 2.3-41) than non-frail patients
D.J. Weaver (2019, world Neurosurgery)	Retrospective review of a prospective database. (III)	23516 (10764:12752)	NS	NSQIP database (2012-2016)	mFI-5	mFI-5 = 0: 8961 (38%) mFI-5 = 1: 10064 (43%) mFI-5≥2: 4471 (19%)	Univariate analysis revealed a stepwise increase in surgical site infection, deep SSI, organ space SSI and wound dehiscence, with increasing of mFI-5. Adjusted multivariate analysis showed that increasing mFI-5 score vs mFI = 0 was associated with higher odds of surgical site infection, deep SSI, organ space SSI and wound dehiscence
K.E. Pierce (2020, Spine)	Retrospective review of a prospective database (III)	191 (38:153)	59	ISSG database (2008-2018)	ASD-FI	Non-frail (ASD-FI<.3): 43,6% Frail (ASD-FI 0.3-.5): 40.8% Severely frail (ASD-FI>.5): 15.6%	There was no significant difference in rate of infections at increasing levels of frailty (*P* = .496)
A.A. Elsamadicy (2021, ths Spine Journal)	Retrospective cohort study (III)	mFI-11 = 0: 2030 (1307:723) mFI-11 = 1: 2319 (1477:842) mFI-11>1: 947 (605:342)	mFI-11 = 0: 55±13.5 mFI-11 = 1: 64±11 mFI-11>1: 66±9.3	NSQIP database (2010-2016)	mFI-11	mFI-11 = 0, non-frail: 38.3% mFI-11 = .18, frail: 43.8% mFI-11≥.27, severely frail: 17.9%	Surgical AEs (superficial, deep and organ space SSI, and wound dehiscence) were significantly greater in the mFI≥2 cohort than the other cohorts, (2.6% vs mFI = 1: 1.8% and mFI = 0: 1.2%, *P* = .022).
K.E. Pierce (2021, clinical spine surgery journal)	Retrospective cohort study (III)	9143 (4023:5120)	59.1	NSQIP database (2005-2016)	mFI-5	Non-frail: (mFI-5<.3): 82.1% Mildly frail: (mFI-5 = .3-.5) 16. % Severely frail: (mFI-5>.5): 1.9%	Surgical site infections did not increase at increasing levels of frailty (*P* = .207)
S. Shahrestani (2021, European spine Journal)	Retrospective cohort study (III)	Frail patients: 5950 (2511:3439) Non-frail patients: 5895 (2505:3390)	Frail patients: 74±5.9 Non-frail patients: 71.1±5.8	NRD (2016-2017)	ICD-10 diagnostic code (according to johns hopkins adjusted clinical group frailty defining diagnosis indicator)	5.5%	Frailty patients had significantly higher rates of infection during primary admission (OR: 6.87, 95% CI:4.55-10.86, *P* < .0001), at 30 days (OR: 1.61, 95%CI: 1.05-2.46, *P* = .027) and at 90 days (OR: 1.51, 95%CI: 1.07-2.16, *P* = .020)
N.V. Shah (2022, journal of clinical Neuroscience)	Retrospective analysis of prospectively collected cohort (III)	2119 (770:1350)	NS (>18 years)	NSQIP database (2008-2016)	mFI-5	mFI-5 = 0: 1020 (48.1%) mFI-5 = 1: 8181 (38.6%) mFI-5≥2: 282 (13,3%)	Individuals with an mFI-5 score of 2 had increased rates of superficial incisional SSI (2.7% vs .7%)
P.G. Passias (2022, Spine)	Retrospective single centre database study (III)	509 (107:402)	59	Single institution database (2014-2018)	m-ASD-FI	Non-frail (m-ASD-FI<7): 50.3% Frail (m-ASD-FI 7-12): 34% Severely frail (m-ASD-FI >12): 15.7%	Frailty and severe frailty were both significantly associated with greater odds for infections. At adjusted analysis severe frailty predict higher odds of infections (OR: 3.5, [1.2-9.7]; *P* = .02),
A. Ton (2022, European spine Journal)	Retrospective cohort study (III)	7088 (3221:3867)	Frail: 74.02±5.84 Non-frail: 74.07±5.56	NRD (2016-2017)	ICD-10 diagnostic code (according to johns hopkins adjusted clinical group frailty defining diagnosis indicator)	Frail: 50% non-frail: 50%	Frail patients encountered higher rates of infection (OR: 2.07, 95% CI 2.04-4.36, *P* < .0001
G. Camino-Willhuber (2023 world Neurosurgery)	Retrospective cohort study (III)	167630 (89675:77955)	59.9±14.1	NSQIP database (2009-2019)	mFI-5	mFI-5 = 0: 40% mFI-5 = 1: 40% mFI-5≥2: 20%	All surgical infectious complications (superficial SSI, deep SSI, organ space SSI and wound dehiscence) increased at increasing of mFI-5 index.
A.A. Elsamadicy (2023, Europeas Spine Journal)	Retrospective cohort study (III)	7500 (2068:5432)	Low HFRS cohort: 60.48 ± 15.25 Intermediate-high HFRS cohort: 65.35 ± 11.09,	The healthcare cost and utilization project’s national impatient sample database (2010-2016)	Hospital frailty risk score (HFRS)	Low (HFRS<5): 53.3% Intermediate-high (HFRS≥5): 46.7%	There was no significant difference in incidence of postoperative procedural infection (*P* = .064)
N. Patel (2023, north american spine society Journal)	Retrospective cohort study (III)	11711 (NS: NS)	NS	NSQIP database (2010-2019)	mFI-5	mFI-5 = 0: 34% mFI-5 = 1: 46% mFI-5≥2: 20%	High frailty scores, defined as 3 +, were not found to be predictive of surgical site infections
N. Patel (2023, global spine Journal)	Retrospective cohort study (III)	156 (83:73)	NS	Single institution database (2012-2021)	mFI-5	mFI-5 = 0: 43% mFI-5 = 1: 38% mFI-5≥2: 19%	Surgical site infections increased at increasing of mFI-5 index without a statistically significant difference

The rate of SSI in different grades of frailty was considered for outcome measure.

When studies involved patients with SSI not solely limited to ASD\DSD patients (such as tumors or fractures), data about patients with ASD\DSD were pooled: if this was not possible, the study was excluded.

### Methodological Quality Assessment of Included Studies

The Quality in Prognosis Studies (QUIPS) tool^30^ was used to assess the methodological quality of the included studies. The quality of each study was reported assessing 6 domains: study participation, study attrition, prognostic factor measurement, outcome measurement, study confounding and statistical analysis and reporting. Each domain can present a low, moderate or high risk of bias: these combined together form an overall risk of bias. For each included study, the total risk of bias was categorized as low risk with ≥4 low-risk domains, moderate risk with <4 low-risk domains, and high risk with ≥1 high-risk domains.

**Figure 2.**
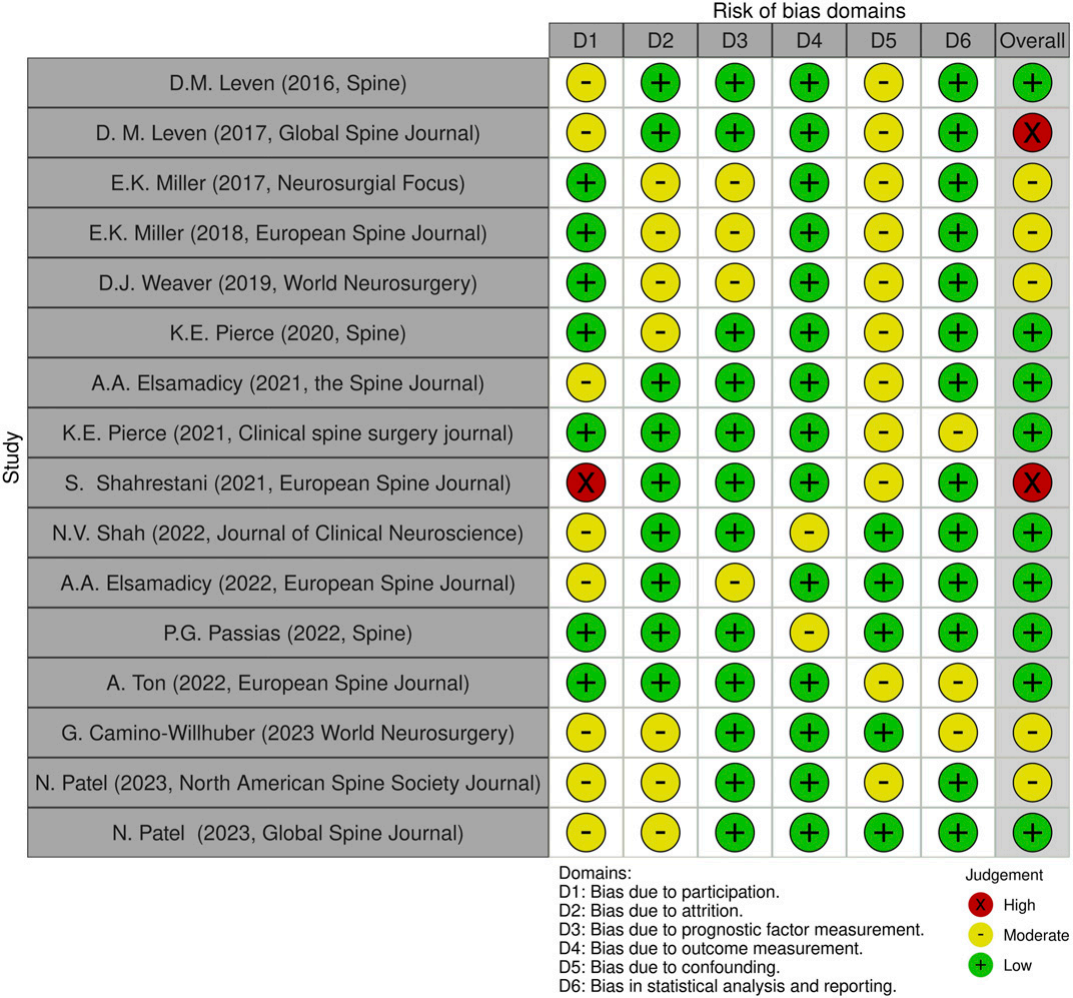
QUIPS Plot of the included studies.

**Figure 3.**
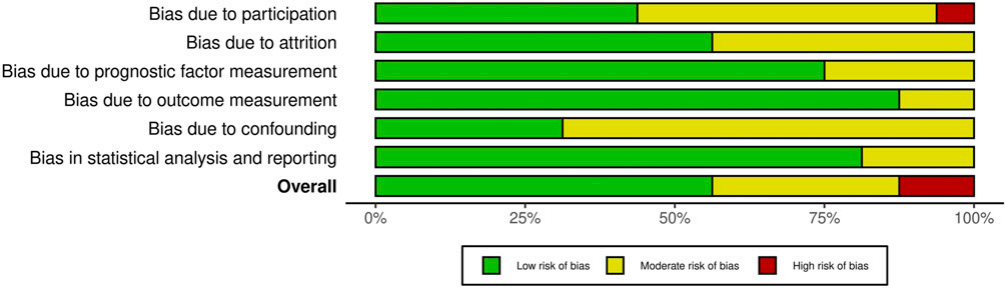
QUIPS plot summary.

As with the evaluation of titles and abstracts, any disagreement was solved by the senior Author (CF). Details on the quality of the studies included are summarized in Figures 2 and 3.

### Statistical Analysis

Meta-analyses were performed when at least three studies were comparable. The analysis was carried out using the log odds ratio with 95% CI and *P* value were used as the outcome measure of effect size. A random-effects model was fitted to the data. The amount of heterogeneity (i.e., tau^2^), was estimated using the restricted maximum-likelihood estimator. In addition to the estimate of tau,^2^ the Q-test for heterogeneity and the I^2^ statistic are reported. In case any amount of heterogeneity is detected (i.e., tau^2^ > 0, regardless of the results of the Q-test), a prediction interval for the true outcomes is also provided. Studentized residuals and Cook’s distances are used to examine whether studies may be outliers and/or influential in the context of the model. Studies with a studentized residual larger than the 100 × (1 - .05/(2 × k))th percentile of a standard normal distribution are considered potential outliers (ie, using a Bonferroni correction with two-sided alpha = .05 for k studies included in the meta-analysis). Studies with a Cook’s distance larger than the median plus six times the interquartile range of the Cook’s distances were considered to be influential. The rank correlation test and the regression test, using the standard error of the observed outcomes as predictor, are used to check for funnel plot asymmetry.

All statistical analyses were conducted with Jamovi version 2.2 (The Jamovi Project, Sydney, Australia) software. *P* value <.05 was considered to be significant.

### Informed Consent and Institutional Review Board Approval

Ethical approval and institutional review board approval were not required because this study would retrieve and synthesize data from already published studies.

## Results

### Included Studies

Initially, a total of 1440 studies were found through electronic search. Before title screening, 2 article were excluded after duplication removal. 1438 Records were screened by title and abstract leading to the exclusion of 1343 records. After screening, 95 studies were assessed for eligibility. The inclusion criteria were not met by 79 studies, such as those that included revision surgery patients, those with traumatic, neoplastic, or cervical spine diseases, or those that reported no or insufficient postoperative SSI data or on frailty rate. Eventually 16 studies^16-19,21-24,31-38^ met the inclusion criteria and were included in the systematic review for qualitative synthesis. Eight studies were considered for quantitative analysis (3 studies regarding frailty and SSI rate when frailty was measured with mFI-11,^16,17,38^ and 5 for frailty and SSI rate when frailty was measured with mFI-5^32-35,37^). (Figure 1).

Nine^22,24,31-36,38^ of the included studies were designed in a retrospective cohort fashion, with either prospectively or retrospectively collected data, and seven^16-20,23,37^ were retrospective analysis of databases studies. In all of the included studies, authors enrolled patients using a variety of databases from various years of recruitment: National Surgical Quality Improvement Project database (NSQIP)^16,17,31-33,35,37,38^ from 2005 to 2019, International Spine Study Group (ISSG)^18,20^ database from 2008 to 2018, European Spine Study Group database^19^ from 2012 to 2013, Nationwide Readmission Database (NRD)^22^ from 2016 to 2017, healthcare cost and utilization project’s national impatient sample database^36^ from 2010 to 2016, and single institution databases^23,35^ from 2012 to 2021. No intervention studies were found in any of the explored databases.

Included studies reported data on a total of 254,482 patients (119,687 females, 47.2%) and the median age at surgery ranged from 43 ± 1.7 to 74 ± 5.9 years.

In all analyzed studies, each group of frail patients in which SSI occurred was matched with a relatively homogeneous group composed by non-frail patients affected by postoperative SSI.

Included studies analyzed both small and large-sized populations and were heterogeneous in the description of frailty evaluation tool and prevalence of frailty (Table 1).

### Risk of Bias Assessment

Two authors (MM and GV) assessed the risk of bias for each study using the QUIPS tool, results are shown in Figure 2.

Most studies^16-19,21-23,31-38^ indicated an overall risk of bias low or moderate (respectively 56.25% and 37.5%, total 93.75%). However, one study^38^ (6.25%) had a high risk of bias, due to the bias caused by participation. Since most studies well-described the outcome measurement with a clear definition of the result, accurate and reliable outcome measurements, and outcome assessment, they demonstrated a low outcome and prognostic factor measurement items (<80%). Furthermore, the confounding measurement and account item was consistently moderate for most studies (68,75%) since the observed influence of prognostic variables on outcome may be skewed by another component linked to the outcome. Other studies (31,25%) performed multiple multivariate analysis to reduce the influence of confounding variables and identify independent risk factors for infection.

### Frailty Evaluation Tools and Prevalence

All included studies evaluated the prevalence of frailty in their population. Six distinct frailty indices were discussed in this review, with emphasis on the most well-known frailty indices: the modified frailty index-11 variables (mFI-11),^16,17,38^ modified frailty index-5 variables (mFI-5),^31-35,37^ adult spine deformity frailty index (ASD-FI),^18-20^ modified adult spine deformity frailty index (m-ASD-FI),^23^ Hospital frailty risk score (HFRS)^36^ and the ICD-10 diagnostic code for frailty^22^; Table 2 provides insights on each of these indices.

**Table 2.** Details of the Principal Frailty Indices Used in Included Studies, mFI-11 = Modified Frailty Index 11 Elements, ASD-FI = Adult Spine Deformity Frailty Index, mFI-5 = Modified Frailty Index 5 Elements, HRFS = Hospital Risk Frailty Score.

mFI-5 Components	mFI-11 Components	ASD-FI Components	HRFS Components
		1) Health deficits documented by physician.	• Dementia in Alzheimer’s disease
		• >3 medical problems	• Hemiplegia
		• Body mass index <18.5 or >30 kg/m^2^	• Alzheimer’s disease
		• Cancer	• Sequelae of cerebrovascular disease
		• Cardiac disease	• Other symptoms and signs involving the nervous and musculoskeletal systems
		• Currently on disability	• Other disorders of urinary system
		• Depression	• Delirium, not induced by alcohol and other psychoactive substances
		• Diabetes	• Unspecified fall
		• Hypertension	• Superficial injury of head
		• Liver disease	• Unspecified haematuria
		• Lung disease	• Other bacterial agents as the cause of diseases classified to other chapters
	• Non- independent functional status	• Osteoporosis	• Other symptoms and signs involving cognitive functions and awareness
	• History of diabetes mellitus	• Peripheral vascular disease	• Abnormalities of gait and mobility
	• History of chronic obstructive pulmonary disease	• Previous blood clot (deep vein thrombosis/pulmonary embolism/stroke)	• Other cerebrovascular diseases
	• History of congestive heart failure	• Smoking status	• Convulsions, not elsewhere classified
• Non- independent functional status	• Hypertension requiring the use of medication	2) Patient-reported (questionnaire, question no.)	• Somnolence, stupor, and coma
• History of diabetes mellitus	• History of percutaneous coronary intervention, cardiac surgery, or angina	• Bladder incontinence	• Complications of genitourinary prosthetic devices, implants, and grafts
• History of chronic obstructive pulmonary disease	• History of myocardial infarction	• Bowel incontinence	• Intracranial injury
• History of congestive heart failure	• Peripheral vascular disease or rest pain	• Deteriorating health this yr (SF-36v2, 2)	• Fracture of shoulder and upper arm
• Hypertension requiring the use of medication	• Impaired sensorium	• Difficulty climbing 1 flight of stairs (SF-36v2, 3e)	• Other disorders of fluid, electrolyte, and acid-base balance
	• Transient ischaemic attack or cerebrovascular accident without residual deficit	• Difficulty driving a car (LSDI, 3)	• Other joint disorders, not elsewhere classified
	• Cerebrovascular accident with deficit	• Difficulty getting dressed (SF-36v2, 3j; LSDI, 1 & 2)	• Volume depletion
		• Difficulty getting in/out of bed (LSDI, 6)	• Senility
		• Difficulty sleeping >6 hrs (ODI, 7)	• Care involving use of rehabilitation procedures
		• Difficulty walking 100 yards (SF-36v2, 3i)	• Unspecified dementia
		• Difficulty w/light activity (SF-36v2, 3b)	• Other fall on same level
		• Feeling downhearted/depressed most of the time (SF-36v2, 9f; SRS-22r, 16)	• Problems related to medical facilities and other health care
		• Feeling tired most of the time (SF-36v2, 9i)	• Vascular dementia
		• Feeling worn out most of the time (SF-36v2, 9g)	• Superficial injury of lower leg
		• General health: Fair/poor (SF-36v2, 1)	• Cellulitis
		• Inability to bathe w/o assistance (SF-36v2, 3j; LSDI, 8)	• Blindness and low vision
		• Inability to cheer up often (SF-36v2, 9c; SRS-22r, 7)	• Deficiency of other B group vitamins
		• Inability to do normal work/schoolwork/housework (ODI, 10; SRS-22r, 9 & 12)	• Problems related to social environment
		• Inability to lift heavy objects (SF-36v2, 3c; ODI, 3)	• Parkinson’s disease
		• Inability to travel >1 hr (ODI, 9)	• Syncope and collapse
		• Inability to walk w/o assistive device (ODI, 4)	• Fracture of rib(s), sternum, and thoracic spine
		• Leg weakness	• Other functional intestinal disorders
		• Loss of balance	• Acute renal failure
		• Not in excellent health (SF-36v2, 11d)	• Decubitus ulcer
		• Personal care dependency (ODI, 2)	• Carrier of infectious disease
		• Restricted activity level (SRS-22r, 5)	• Streptococcus and staphylococcus as the cause of diseases classified to other chapters
		• Restricted social life (ODI, 8; SRS-22r, 14 & 18)	• Ulcer of lower limb, not elsewhere classified
			• Other symptoms and signs involving general sensations and perceptions
			• Duodenal ulcer
			• Hypotension
			• Unspecified renal failure
			• Other septicaemia
			• Personal history of other diseases and conditions
			• Respiratory failure, not elsewhere classified
			• Exposure to unspecified factor
			• Other arthrosis
			• Epilepsy
			• Osteoporosis without pathological fracture
			• Fracture of femur
			• Fracture of lumbar spine and pelvis
			• Other disorders of pancreatic internal secretion
			• Abnormal results of function studies
			• Chronic renal failure
			• Retention of urine
			• Cerebral infarction
			• Calculus of kidney and ureter
			• Mental and behavioural disorders due to use of alcohol
			• Other medical procedures as the cause of abnormal reaction of the patient
			• Abnormalities of heartbeat
			• Unspecified acute lower respiratory infection
			• Problems related to life-management difficulty
			• Other abnormal findings of blood chemistry
			• Personal history of risk-factors, not elsewhere classified
			• Open wound of forearm
			• Depressive episode
			• Spinal stenosis
			• Disorders of mineral metabolism
			• Polyarthritis
			• Other anaemias
			• Other local infections of skin and subcutaneous tissue
			• Nausea and vomiting
			• Other noninfective gastroenteritis and colitis
			• Fever of unknown origin

The frailty prevalence measured using mFI-11 ranged from 3% to 17.9%, these values were inferior to those measured with mFI-5 (13% to 20%). The frail rose between 33.8% and 40.8% when measured by the ASD-FI, while that of the severely frail was described between 14.4% and 20.2%.

### Postoperative SSI Rate and Association with Frailty

All included studies assessed as primary outcome of interest the prevalence of major perioperative medical and surgical complications, and their association with different levels of frailty. A major complication was defined as that substantially changed the expected path to recovery and was potentially life-threatening, required reoperation, or caused permanent injury. These complications included intraoperative vascular, visceral, or neurologic injury, SSI, infections, junctional disease\failure and similar. Medical complications included those unrelated to surgical technique, including stroke, deep venous thrombosis, pulmonary embolus, pneumonia, and urinary tract infection.

Meta-analysis was performed on studies in whom data regarding patients with different levels of frailty, assessed using the same frailty index, and occurrence of postoperative SSI could be pooled; this was possible for five studies^31-35,37^ that quantified frailty with mFI-5, and for 3 studies^16,17,38^ quantifying frailty using mFI-11.

The mFI-5 is the simpler modification of the mFI-11, obtained by removing six variables from the mFI-11. Despite this, in Kweh et al.^39^ study the mFI-5 and mFI-11 were equally effective predictors of postoperative morbidity and mortality in this population. The brevity of the mFI-5 is advantageous in facilitating its daily clinical use. This permitted us to perform a quantitative analysis in very similar series.

In the mFI-5 case, the observed log odds ratios ranged from .2293 to 1.4404, with all estimates being in favor of SSI. The estimated average log odds ratio based on the random-effects model was = .3991 (95% CI: .2678 to .5305).

**Figure 4.**
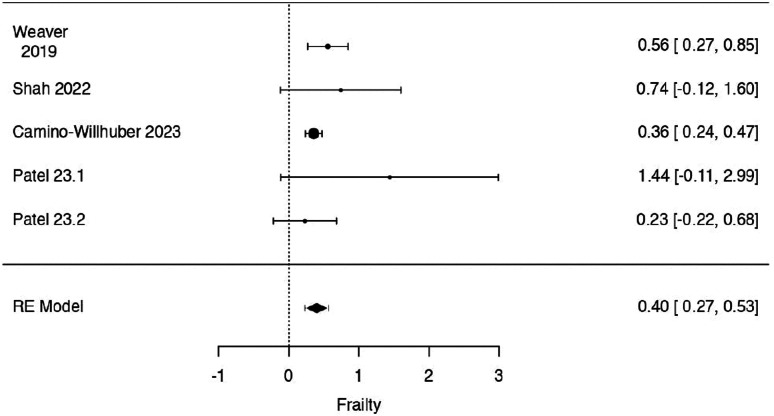
Forrest plot representation of the metanalysis for mFI-5.

Therefore, significant difference was found between frail, intended as mFI-5 ≥2, and non-frail patients in postoperative SSI rate at metanalysis (z = 5.9547, *P* < .0001) (Figure 4).

Regarding mFI-11, the observed log odds ratios ranged from .6620 to .8146, with all of estimates being in favor of SSI. The estimated average log odds ratio based on the random-effects model was = .7539 (95% CI: .3685 to 1.1394).

**Figure 5.**
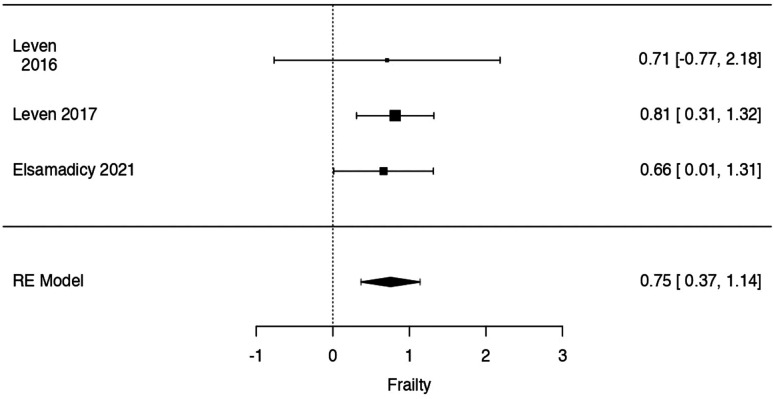
Forrest plot representation of the metanalysis for mFI-11.

Therefore, significant difference was found between frail, intended as mFI-11 ≥ .27, and non-frail patients in postoperative SSI rate at metanalysis (z = 3.8334, *P* = .0001) (Figure 5).

Weawer et al.^37^ in their NSQIP cohort queried for DSD, described at univariate analysis a stepwise increase in SSI, and also in deep SSI, organ space SSI and wound dehiscence, with increasing of mFI-5 values. Adjusted multivariate analysis showed that increasing mFI-5 score vs mFI = 0 was associated with higher odds of SSI, deep SSI, organ space SSI and wound dehiscence. Similar results were described by Leven et al^16^ and Elsamadicy et al^38^ in similar cohorts, where the rate of wound complications due to SSI increased in a stepwise fashion with increasing of mFI-11 (*P* < .02), yet the frail patients cohorts were significantly older than the non-frail cohorts.

In a single institution’s database of DSD patients, Patel et al.^34^ assessed the predictive power of the mFI-5 scores and major perioperative complications, including SSI. The authors found that high levels of frailty were independent predictors of reoperation and associated readmission when compared to the non-frail group, with SSI rising as mFI-5 values increased, however not statistically significantly. These results were confirmed by another study^35^ from the same author, where SSI did not increased with increasing level of mFI-5.

Other included studies specifically investigated complications in frail ASD patients. Shah et al.^32^ analized the utility of mFI-5 in predicting postoperative complications, including SSI, after surgery for ASD using the American College of Surgeons NSQIP database, with the hypothesis that a higher mFI score would be predictive of higher rates of complications. As expected, individuals with an mFI-5 score of 2 or more had increased rates of SSI (4.2% vs .7%) reaching statistical significance at univariate analysis. Even so, the outcome variables were limited to a 30-day follow-up, limiting other potential complications.

Two studies by Miller et al.^18,19^ sought to demonstrate the ability of ASD- FI to predict adverse postoperative outcomes, querying two different large databases (ESSG and ISSG databases) for ASD surgical patients. In both studies, on univariate analysis the severely frail patients had higher odds of wound infection compared to non frail patients (OR 4.3 95% CI 1.0-18, *P* = .04; and OR 8.4; 95% CI 1.6-7.6, *P* < .001). Moreover, these associations were confirmed significant on multivariate logistic analysis for counfounding variables in both studies.

Despite the above-mentioned studies, contradictory results have also been described for ASD patients. In Pierce et al study^20^ the retrospective review of the International Spine Study Group (ISSG) database queired for ASD patients, did not show significant difference in rate of infections at increasing ASD-FI scores (*P* = .496). Same results were advocated by Elsamadicy et al,^36^ where postprocedural SSI did increased with increasing level of frailty, but without reaching statistical significance.

## Discussion

In the present metanalysis we sought to determine the influence of frailty on postoperative SSI in ASD or DSD patients. The frailty prevalence was quite consistent among the included studies, with values ranging from 3% to 40% depending on the index used for measuring frailty. When measured with mFI-11 or mFI-5, frailty patients showed a statistically significant difference on SSI rate compared to non-frail patients, with mFI-5 showing a slightly greater significance than mFI-11.

While adverse events are not always predictable, factors such as patient characteristics, comorbidities, surgical technique, procedural modifications, and surgeon skills have a substantial influence. Frail patients are prone to adverse events after spine surgery.^16-19,22-24,31-35,37,38^ As the frailty severity increases, the rate of major perioperative adverse events, readmission for revision surgery, and mortality increases, which has been shown across several surgical and orthopedic subspecialities.^40-43^ This association persisted in multivariate analyses, underlining that the influence of frailty is independent from other risk factors, such as surgical procedure or invasiveness, or operative time.^17,18^

In a recent analysis of frail patients undergoing arthroscopic rotator cuff repair, increasing levels of frailty were predictive for medical complications, hospital admission and length of stay. For each point of increase in mFI-5 score, the risk for a medical complication increased by 66%, readmission by 52%, and adverse discharge by 45%. In addition, the mFI-5 was the strongest predictor for mortality, with the risk more than doubling for each increase in mFI-5 point.^44^

SSI is a severe complication of spinal surgery, with an incidence that ranges between .2 and 16%.^13,14^ It can be difficult to treat, resulting in repeated debridement, prolonged antibiotic therapy and potential disability.^45^ Therefore, analyzing and recognizing risk factors is a critical step when evaluating patients for DSD or ASD surgery, particularly as these procedures are overwhelming elective.

The presented metanalysis showed a statistically significant difference between frail, when utilizing either mFI-5 or mFI-11, and non-frail patients in postoperative SSI rate.

The statistical significance of these differences can be highlighted by the relatively consistent direction of the outcome showed among the included studies. Particularly, some of these^16-18,24,32^ suggest that a large percentage of early debridement surgery were caused by SSI, with frailty scores as clear predictors. Leven et al^17^ in their ASD cohort found that as mFI-11 increased from 0 to .29, wound complications due to SSI increased from 3% to 10% and reoperation from 5% to 15%. In another study^32^ on over 2000 ASD surgically treated patients, as mFI-11 increased from 0 to 3, superficial and deep SSI and wound dehiscence, increased in parallel. This trend was confirmed also in DSD surgery, with two studies analyzing about 6000 patients,^16,24^ where frail patients had significant higher odds of developing postoperative SSI to non-frail patients.

Similar findings were described also in trauma patients over the age of 60 years old, where a direct association between frailty and rates of SSI, any infection and mortality were confirmed.^46^

These complications have a massive impact on long term outcomes and on health care costs for reimbursement based on quality and value metrics and avoiding them is the primary goal for all spine surgeons and patients. Hannah et al.^47^ gave proof of this belief, stating that frail patients had markedly greater risk of total complications (28.4 times higher), greater average length of stay (33.3 vs 2.9) and direct cost ($80,410 vs $16,187) compared to non-frail patients. Moreover, analyzing the impact of frailty on adverse events is an important factor to consider when implementing Enhanced Recovery After Surgery (ERAS) programs that are gaining more and more importance in different medical subspecialities and in spine surgery.^48,49^

Despite many historical indices attempted to quantify frailty into a single scoring index, there has only been partial success in applying them to clinical practice. However, some have been described and validated in literature. The results of the present metanalysis show that indices based on accumulation of deficit model suggested by Rockwood^50^ (mFI-11 and 5, ASD-FI, HFRS) were employed in almost all of the included studies.

These indices provide several advantages. Firstly, are readily accessible tool for surgeons and clinicians to objectively perform a risk stratification analysis in preoperative candidates.

This enables efficient informed preoperative counselling to occur and provide opportunity for prophylactic patient optimization, with nutritional status assessment and supplementation, or with physiotherapy for muscle loss, or with medical management of comorbidities.^51,52^

Furthermore, the clinical benefit of these tools is that their components are easily obtained during clinical history acquisition and physical examination and can be used by the surgeon with simple threshold of a limited number of variables to instantly group patients into a low or high risk of perioperative adverse events.^53,54^

Ultimately, one of the most significant advantages is that frailty index was found to be stronger prognostic predictor than age or ASA score in several studies.^17,55^

This might be attributed to the fact that age and ASA score are fairly nonspecific parameters, highly variable between individuals. Indeed, as ageing is considered as a perioperative risk factor, chronologic age has a very limited significance as elderly individuals could still healthy, and prior studies have shown that 75% of patients over 85 are not frail.^56^ However, some patients in this age group have extensive medical comorbidities and their biologic age is considerably frail.

This work does not come without limitations. First, all the included studies were performed in a retrospective fashion, though most of them collected data prospectively. Therefore, the level of evidence of the obtained results are inevitably suboptimal.

As stated in literature, we think that risk factors could be more or less relevant depending on the surgery invasiveness, as an example in “short” vs more invasive spinal procedure. However, investigation of other risk factors, was not the purpose of our study, we limited to a quantitative exploration of the data, without considering the specific weight of surgical invasiveness. Moreover, as stated by above, as the frailty severity increases, the rate of major perioperative adverse events, like SSI, increased and this association persisted in multivariate analyses, underlining that the influence of frailty is independent from other risk factors.

Moreover, most studies queried large databases for gathering their experimental cohort. Although large databases permit to gather large number of patients, they are quired via ICD-9 and CPT codes to identify diagnosis, procedure, comorbidities, and perioperative adverse events. This could have generated underreporting or heterogenous data, seen that every hospital filling the database could consider different factors for the same diagnosis or complication. In our study, we have acknowledged and addressed this concern by distinguishing between qualitative and quantitative evaluations. Although some of the included studies may have accessed similar databases for overlapping time periods, these instances were solely considered within the qualitative assessment of our topic, where the overlapping of patient data does not translate into a quantitative discrepancy and still transmits the importance of frailty influence on SSI.

For the quantitative analysis, the included studies either consulted different databases or the same databases but for distinct identification codes and spans of years, all of which fall under the ASD or DSD categories. This may have reduced the potential for overlap, exerting a moderate impact on our findings and on the assessment of frailty’s influence on SSI.

The indices analyzed by the included studies show limitations as well. One of these is that none considers laboratory or radiographic factors that could influence frailty, such as C-reactive protein, interleukins or radiographic indices of sarcopenia\osteopenia. Moreover, mFI5 and 11, don’t measure several characteristics of frailty, such as weight loss, slow walking speed, exhaustion, weakness and low physical activity.

Despite these limitations, this is the first meta-analysis to specifically investigate the impact of frailty, measured by specific indices, on occurrence of SSI. We found a relevant statistical difference between frail and non-frail patients in SSI occurrence rate. This is a relevant finding, as the ageing of population increases alongside with spine surgery procedures for ASD and DSD, a better understanding of risk factors may advance our ability to treat patients and improve their quality of life while minimizing the occurrence of SSI.

Further studies are needed to increase the level of evidence on this topic and to investigate more specific frailty indices that were not possible to include in a meta-analysis.
